# Immune Evasion Strategies during Chronic Hepatitis B and C Virus Infection

**DOI:** 10.3390/vaccines5030024

**Published:** 2017-09-01

**Authors:** Ana Maria Ortega-Prieto, Marcus Dorner

**Affiliations:** Section of Virology, Department of Medicine, Imperial College London, London W2 1PG, UK; a.ortega-prieto@imperial.ac.uk

**Keywords:** Hepatitis B virus, Hepatitis C virus, innate immunity, adaptive immunity, immune evasion

## Abstract

Both hepatitis B virus (HBV) and hepatitis C virus (HCV) infections are a major global healthcare problem with more than 240 million and 70 million infected, respectively. Both viruses persist within the liver and result in progressive liver disease, resulting in liver fibrosis, cirrhosis and hepatocellular carcinoma. Strikingly, this pathogenesis is largely driven by immune responses, unable to clear an established infection, rather than by the viral pathogens themselves. Even though disease progression is very similar in both infections, HBV and HCV have evolved distinct mechanisms, by which they ensure persistence within the host. Whereas HCV utilizes a cloak-and-dagger approach, disguising itself as a lipid-like particle and immediately crippling essential pattern-recognition pathways, HBV has long been considered a “stealth” virus, due to the complete absence of innate immune responses during infection. Recent developments and access to improved model systems, however, revealed that even though it is among the smallest human-tropic viruses, HBV may, in addition to evading host responses, employ subtle immune evasion mechanisms directed at ensuring viral persistence in the absence of host responses. In this review, we compare the different strategies of both viruses to ensure viral persistence by actively interfering with viral recognition and innate immune responses.

## 1. Introduction

Chronic viral hepatitis (CVH) infections are a major global public health concern with more than 240 million and 70 million people infected with hepatitis B virus (HBV) and hepatitis C virus (HCV), respectively [1,2]. Both viruses persist within infected hepatocytes in the liver, where they eventually result in the development of liver fibrosis, cirrhosis and hepatocellular carcinoma [3]. Despite the similarity in disease progression and target cells, HCV and HBV have developed complex but nearly opposing strategies of ensuring persistence within infected individuals. While HCV infection usually results in vigorous innate immune activation during infection, the clinical symptoms of acute infection are often subclinical and may go unnoticed [4,5]. In contrast, acute HBV infection is usually easily detectable through the accompanying symptoms [6]. However, HBV itself seems to engage far fewer innate and inflammatory pathways, compared to HCV infection.

Both, acute and chronic infection by HCV and HBV may eventually result in the development of a life-long, chronic infection in 80% and 5% of adults, respectively. However, HBV infection during childhood may result in chronic infection in as many as 90% of infected, suggesting a strong contribution of immune maturation on the development of persistent infection [7]. In the case of spontaneous clearance of HCV or HBV infection, a potent adaptive immune response is clearing infected hepatocytes, resulting in temporary liver injury, as determined by elevated levels of alanine aminotransferases (ALT) [8]. Viral antigen subsequently is completely eliminated and, once cleared results in life-long, sterilizing immunity. In the case of HCV infection, spontaneous clearance has been strongly linked with innate immune activation and interferon responses [9].

Both, HBV and HCV infect hepatocytes of the liver via receptor-mediated endocytosis, where they initiate replication. During the initial interaction with their host cell, both, HBV and HCV are exposed to a number of pattern recognition receptors (PRR), which form the first line of defense against infections.

HCV infection is restricted to the cytoplasm, where it reorganizes parts of the endoplasmic reticulum to form the membranous web and the replicase compartment [5]. Ranging from the initial translation of the HCV polyprotein and RNA replication to viral assembly of virions on lipid droplets, HCV RNA and proteins are constantly prone to triggering innate cellular immune responses, especially the uncapped 5′ internal ribosomal entry site and its extensive secondary structure, resulting in the formation of double-stranded RNA intermediates are potent pathogen-associated molecular patterns (PAMP). To circumvent cellular sensing, HCV has evolved several intricate mechanisms, by which it prevents the host from initiating interferon responses.

In contrast, HBV capsids are directly shuttling into the nucleus of infected cells before disassembly and release of the relaxed-circular (rc) HBV DNA genome [8]. During subsequent complex, and thus far poorly understood mechanisms, host factors are recruited in order to remove the covalently-bound HBV reverse transcriptase from rcDNA and repair the incomplete (+)-strand, resulting in covalently closed, circular (ccc)DNA, which forms the basis of all de novo produced HBV. Unique to HBV, its cccDNA genome is recruiting host-derived histones, forming, what is commonly known as HBV minichromosome. All viral transcripts are processed by host polymerase II, capped and polyadenylated before translation. One of these transcripts, the overlength pgRNA, is encapsidated prior to reverse transcription, effectively hiding or shielding most of the HBV life-cycle from immune detection.

Whereas HCV replication during acute and chronic infection is uniformly recognized by the adaptive immune system, HBV infection is divided into distinct clinical stages, which are associated with varying degrees of immune activation and subsequent liver injury [10]. Early after infection, HBV replicates nearly uncontrolled within the infected liver (termed HBeAg-positive infection or “immune tolerant” phase), resulting in up to 80% of hepatocytes becoming infected and serum HBV DNA titers of more than 20,000 IU/mL [11]. Immune activation and liver injury are negligible during this stage of infection; however, more evidence is gathering that HBV actively subverts innate immunity during this phase of infection. Once the immune system recognizes HBV infection (termed HBeAg-positive hepatitis or “immune reactive” phase), which in many cases occurs only years after infection, a strong CD8 T cell response results in viral suppression accompanied by marked liver injury [11]. To date, it remains poorly defined, as to what triggers the transition from HBeAg-positive infection to hepatitis. This phase of immune clearance is followed by seroconversion from HBeAg positive to anti-HBe antibodies. This is often accompanied by particular mutations within the HBV pre-core or basal core promoter, resulting in reduced or absent HBeAg secretion [12]. During this phase, referred to as HBeAg-negative infection or “inactive carrier” state, serum HBV DNA levels are below 2000 IU/mL and persistently normal ALT levels. Following the establishment of HBeAg-negative infection, HBV may periodically reactivate (termed HBeAg-negative hepatitis), resulting in sporadic flares of serum HBV DNA elevations accompanied by ALT level elevations [13].

Common to both, HBV and HCV infection, is a further subversion of adaptive immune responses during chronic infection [8]. Both pathogens result in a terminally exhausted T cell phenotype, with most HBV- or HCV-specific CD8 T cells being anergic and unable to mount an effective polyfunctional response [14]. In terms of humoral responses, HBV and HCV have evolved different means of subverting B cell responses. Despite the potent ability of HBsAg to elicit neutralizing antibody responses, HBV replication produces an enormous quantity of sub-viral particles, which compete with the infectious virions for antibody binding, thus incapacitating any antibody-based neutralization. HCV infection on the other hand generally does not result in the development of sterilizing antibody-based immunity, which is due to HCV virions mimicking very low-density lipoproteins (VLDL) to evade antibody targeting and virus spreading through tight junctions between two hepatocytes, where they are immune to antibody neutralization.

Here, we give an overview of how HBV and HCV interact with the hosts’ innate and adaptive immune system and the mechanisms evolved in order to ensure persistence.

## 2. Host Responses to HBV and HCV Infection

### 2.1. Cell-Intrinsic Innate Immune Responses

**Figure 1:**
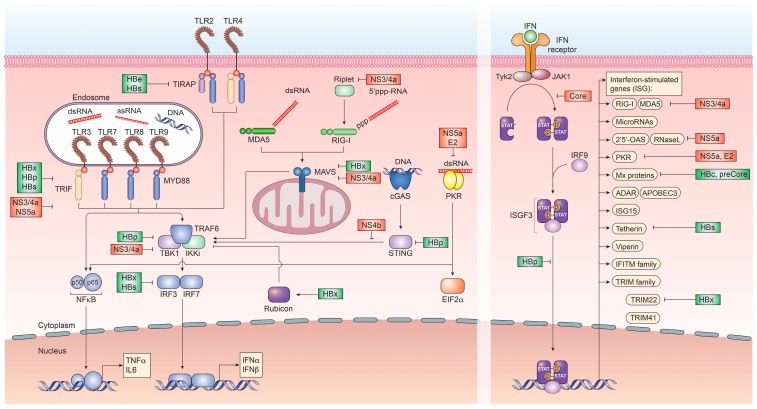
Recognition of hepatitis B virus (HBV) and hepatitis C virus (HCV) by pattern-recognition receptors and immune evasion strategies. Innate immune signaling pathways involved in the recognition of HBV and HCV infection and the subsequent induction of interferon and interferon-stimulated genes. Several viral proteins of HCV (red) and HBV (green) interfere at various stages with these pathways.

**Figure 2:**
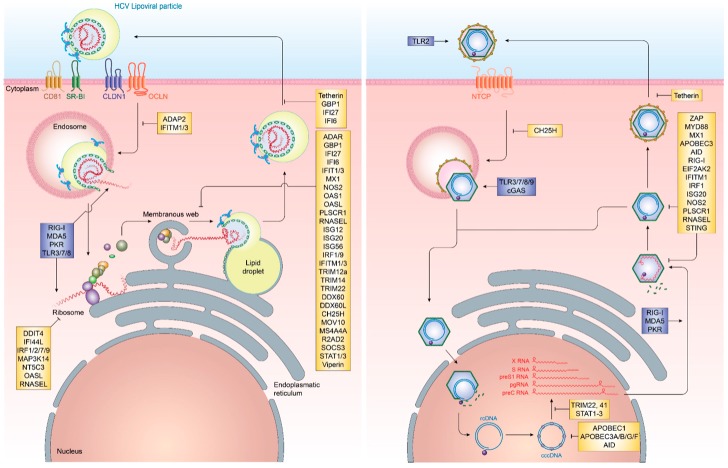
The life cycle of HCV and HBV and interferon effector molecules targeting different steps in the viral infection process. HCV (left) and HBV (right) life cycles including pattern recognition receptor targets (blue) and interferon-stimulated genes (yellow) affecting viral entry, replication and egress.

Cell-intrinsic innate immune responses are among the first line of defense against incoming infectious diseases. Pattern-recognition receptors (PRR) located on the cell membrane, intracellular vesicles or within the cytoplasm recognize conserved pathogen-associated molecular patterns (PAMP), including foreign proteins, lipids and nucleic acids, resulting in the initiation of signal transduction pathways, culminating in the production of pro-inflammatory cytokines and interferons (IFN) [15] (Figure 1). Three distinct classes of IFN can be produced by different cell types, type I (IFNα, IFNβ), type II (IFNγ) and type III (IFNλ1, IFNλ2, IFNλ3, IFNλ4) [16,17]. Once produced, they are secreted and bind to their cognate receptors (IFNAR1/2 for type I IFN, IFNGR for type II IFN and IL28RA/IL10R2 for type III IFN) in an autocrine or paracrine manner and trigger the JAK/STAT signal transduction pathway. For type I and III IFN, this, in turn, results in the up-regulation of hundreds of interferon-stimulated genes (ISG), which exert antiviral functions [18]. These ISG can be broadly classified into several categories. In the case of HCV, these include ISG with direct antiviral function, including inhibitors of HCV entry, translation, replication and release as well as ISG, which are part of the active sensing and transcriptional activation system or negative regulators of innate immune activation (Figure 2 and Table 1). There are numerous ISGs that have been identified playing a role during the HCV viral life cycle, more extended information can be found in the review published by Bartenschlager and colleagues [19]. Expression of these ISG is mainly aimed at restricting the spread of viral infections to allow for the induction of cellular innate and adaptive immune responses. In cases, where viral replication cannot be controlled by the antiviral effector function of ISG, they can additionally result in cell death of infected cells, in order to restrict viral spread.

In contrast to HCV infection, HBV infection does not elicit notable innate immune activation through pro-inflammatory cytokines and IFN. Remarkably, early studies in chimpanzees and humans have demonstrated that HBV infection does not induce any gene expression signature characteristic for IFN or ISG, resulting in HBV to be known as “stealth” virus [87,88,89]. This is further supported by a recent study based on the use of in vitro models and liver chimeric mice, which has shown an absence of IFN responses upon stable HBV replication. Additionally, it was observed that cells previously infected with HBV and stimulated with inducers of IFN signaling successfully mounting a downstream immune response in the presence of active HBV infection. This supports that HBV does not actively evade immune recognition [90]. It, however, remains poorly understood as to why host innate immune responses fail to recognize HBV infection or what mechanism ultimately results in their activation later during infection. Nevertheless, several ISGs have been implicated in the potential control of HBV infection at various steps in its life cycle. Recently, cholesterol-25-hydroxylase (CH25H) was identified as an ISG inhibiting HBV entry through its receptor, Na^+^-taurocholate cotransporting polypeptide (NTCP) [31]. Once HBV cccDNA is formed, members of the nuclear deaminase family, including apolipoprotein B mRNA editing enzyme, catalytic polypeptide-like (APOBEC)3A, APOBEC3G, and AID have been demonstrated to deaminate cccDNA and target it for degradation [22,23,28]. cccDNA itself, due to its chromatinized structure, is additionally regulated through epigenetic mechanisms, most notably through STAT1 and STAT3, which directly bind to cccDNA and regulate its transcriptional activity [68]. Tripartite motif-containing (TRIM)22 is an additional ISG, which has recently been demonstrated to interfere with the transcriptional activity of HBV cccDNA through direct binding to the HBV core promoter [81]. Other TRIM family members have also been demonstrated to restrict HBV transcription, including TRIM41, which transactivates an E3 ubiquitin ligase to inhibit HBV enhancer elements I and II [83]. The PRR RIG-I has been shown to have a dual antiviral effect on HBV pgRNA. In addition to sensing pgRNA and inducing expression of type III IFN, it counteracts the interaction of HBV polymerase with the 5′-ε region, suppressing viral replication [35]. ISGs additionally act on a posttranscriptional level on HBV pgRNA, including the zinc finger antiviral protein (ZAP), which binds to HBV RNAs and decays it through its ZRE sequence domains [85]. Also, myeloid differentiation primary response protein 88 (MyD88) is able to accelerate the decay of viral pgRNA [60] and myxoma resistance protein 1 (MxA) inhibits the encapsidation of pgRNA [58,91] (Figure 2 and Table 1).

Notably, type I IFNs, especially IFNα, have been extensively used in the past in the treatment of chronic HCV and HBV infection [92]. Even though the systemic administration of IFNα is associated with significant side effects and results in generally poor response rates (e.g., fewer than 50% therapeutic success in HCV infection), it is to date the sole therapeutic intervention, which may result in a functional cure of HBV infection through HBsAg seroconversion [93]. Analysis of genetic factors associated with HCV chronicity have revealed the strong association of IFN, specifically type III IFN with not only treatment response but also with the spontaneous clearance of HCV infection. In particular, human BDCA3+ DCs, mainly allocated in the liver, are able to sense HCV in a CD81-, endosome-, and TRIF-dependent manner and generate high quantities of IL-28B/IFN-λ3. This particular subset of dendritic cells has a superior response to HCV in patients with the major genotype (TT) for the SNP rs8099917 [94]. Genome-wide association studies (GWAS) furthermore revealed that a number of single nucleotide polymorphisms within the locus of type III IFNs are strongly associated with HCV persistence [9,95,96,97]. Later, it was identified that one of the SNPs, variant ss469415590, is forming a thus far unknown IFN, IFNL4, which is resulting in poor treatment outcome and little spontaneous clearance [98], indicating that the role of IFN in viral infections is more complex than originally anticipated. Furthermore, an association of the presence or absences of IFNL4 and other viral infections, including HBV, have still to be confirmed.

### 2.2. Innate Immune Responses

**Figure 3:**
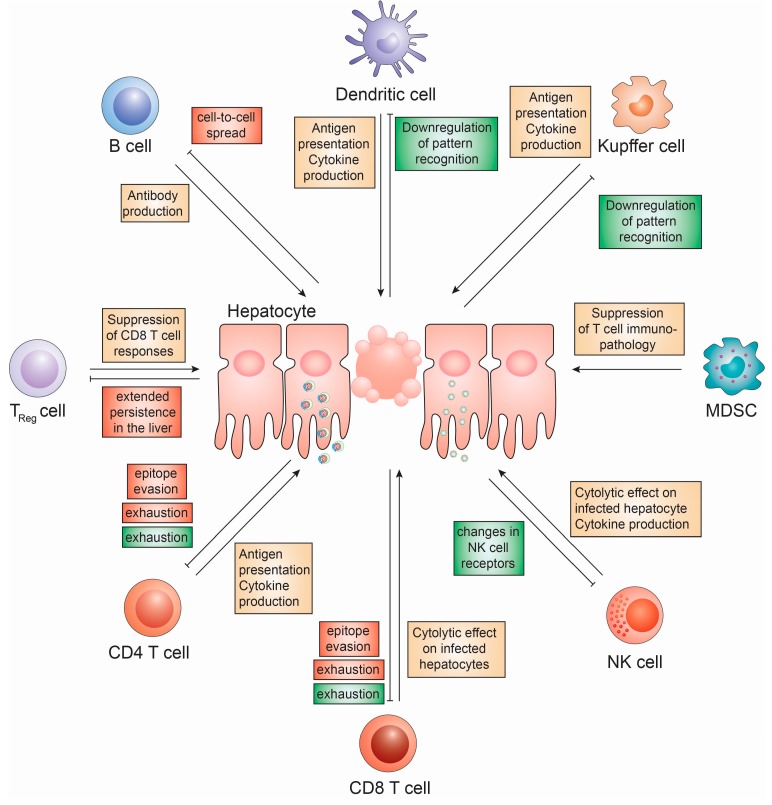
Cell based innate and adaptive immune responses to HBV and HCV infection. The role of innate immune cells (Kupffer cells, myeloid-derived suppressor cells (MDSC), NK cells and Dendritic cells) and adaptive immune cells (B cells, CD4 T cells, CD8 T cells and regulatory T cells) on HBV and HCV infection and mechanisms devised by HCV (red) and HBV (green) to subvert these.

Cell-based innate immune responses against HBV and HCV have been extensively studied in the past years. These include Natural Killer (NK) cell, NKT cells, monocyte/macrophage, dendritic cells as well as specialized myeloid subpopulations (e.g., myeloid-derived suppressor cells) (Figure 3).

#### 2.2.1. NK Cell Responses in Chronic Viral Hepatitis

NK cells, which can be divided into CD56^bright^, IFN**γ**-producing, and Th1-priming, and CD56^dim^, fully mature and highly cytotoxic cell subsets play an important role in early innate immune responses [99]. Since their activation and NK cell-mediated killing is generally independent of prior sensitization, their activation is tightly regulated. This involves a plethora of killer immunoglobin-like receptors (KIR) the lectin-like receptors [100]. NK cell activation, in general, is driven by “missing self” antigen and NK cells are inhibited by the presence of major histocompatibility complex (MHC) class I molecules on target cells, preventing them from killing. Upon loss of MHC class I, which is often down-regulated during viral infections, NK cells kill the target cell via direct cytotoxicity. Both, KIR and MHC genes exhibit significant genetic diversity, resulting in KIR predominantly recognizing specific subgroups of MHC class I alleles. Furthermore, KIR may be inhibitory or activating, furthermore adding complexity to NK cell-mediated killing [101]. NK cells represent a major cell population in the liver, where they are enriched to approximately 30% of liver-resident lymphocytes [102].

Although in vitro data suggest that NK cells can effectively kill HCV-infected replicon cells, it also has been demonstrated that direct contact of NK cells with hepatocytes may impair their killing capabilities and their IFN**γ** response by thus far poorly understood mechanisms [103,104]. Furthermore, it has been suggested that hepatocytes are relatively resistant to perforin- and granzyme-mediated cytotoxicity [105]. During chronic HCV infection, NK cells remain in a state of chronic activation, thus contributing to the progression of liver fibrosis in HCV infection [106].

Elegant experiments in the HBV-transgenic mouse model have identified NK1.1^+^CD3^−^ NK cells as the major constituent of inflammatory infiltrates in the liver [107]. This large number of NK cells in HBV infection could contribute to the liver injury, which is observed during HBV infection. Additionally, hepatocyte apoptosis has been implicated as the main event in the initiation of hepatic inflammation and HBV-infected apoptotic cells result in an immunogenic rather than tolerogenic immune response [108]. In particular, TRAIL, which is expressed specifically on NK cells in the liver of HBV-infected patients, has been reported to correlate with liver damage [109]. TRAIL is particularly highly expressed on CD56^bright^ NK cells, which are preferentially expanded in the liver [110]. Another important NK cell-mediated hepatocyte apoptosis pathway is initiated by Fas, which plays a particularly important role in DC-activated NK cells [111]. Finally, NK cells are a potent source of the pro-inflammatory cytokine TNF, which is greatly enhanced during chronic HBV infection and has been shown to inhibit HBV replication via disruption of capsid integrity [112,113].

#### 2.2.2. NKT Cell Responses in Chronic Viral Hepatitis

NKT cells, which are greatly enriched in the liver, bridge the gap between innate and adaptive immune responses. They are T cells, which, in contrast to conventional T cells, recognize lipid-based antigen via the MHC-like molecule CD1d [114]. Once activated, NKT cells are able to secrete a diverse range of cytokines, which contributes to the activation of other immune cells. NKT cells are divided into two distinct families, type I “invariant” NKT cells, which harbor the Vα24-Jα18 invariant TCR α chain, recognizing α-galactosylceramide (α-GalCer) and type II “non-invariant” NKT cells, which express a range of TCR and bind various lipid antigen [114]. Both these subclasses of NKT cells have been shown to be activated during HBV infection, resulting in them expressing IFNγ [115].

#### 2.2.3. Kupffer Cell Responses in Chronic Viral Hepatitis

Kupffer cells (KCs), the liver-resident macrophage type, which usually plays an essential role in regulating liver immune functionality and tolerance, are long-lived and abundant in the liver, constituting approximately 15–20% of the total liver cell population [116]. Their roles range from regulating the tolerogenic environment in the liver, suppressing T cell activation to orchestrating immune responses. KCs are professional antigen-sensing cells and express a variety of PRR, rendering them capable of secreting large amounts of reactive oxygen species (ROS), type I IFN and pro-inflammatory cytokines [117].

In contrast to HBV DNA replication, which is occurring hidden within capsids, HCV replication generates a plethora of pathogen-associated molecular patterns (PAMP). To date, the role of KCs as host cells for HCV in addition to hepatocytes remains controversial [117]. If infected, KCs could play an important role in recognizing early HCV infection [117]. During chronic HCV infection, KCs exhibit massively enriched MHC class II expression levels, which has been associated with the presence of IFNγ, which may likely be produced by NK cells [118]. Usually, this MHC class II expression facilitates priming of CD4 T cells, however, KCs have the ability to cross-present viral antigen to CD8 T cells, presenting an effective means for KCs to prime CD8 T cell responses in the infected liver [119]. A number of studies, however, indicate aberrant KC functions in HBV- and HCV-infected individuals, which may be a result of HBV-mediated TLR2 down-regulation on KCs leading to reduced immune surveillance [118,120,121] (Figure 2). Additionally, KCs have been shown to secrete reduced amounts of TNFα in the presence of HBV antigen, which has previously been demonstrated to affect HBV replication by disrupting capsid integrity through activation of NFκB [113,122]. Recently, it has been shown that exposure of primary monocyte-derived macrophages to high titers of HBV induced expression of inflammatory cytokines. In contrast, these cells are unable to sense HBV, when low titers were used [90].

#### 2.2.4. The Role of Myeloid-Derived Suppressor Cells in Chronic Viral Hepatitis

Recently, bone marrow-derived myeloid-derived suppressor cells (MDSC) have been reported to play a fundamental role in regulating liver injury during chronic HBV as well as HCV infection [123]. Specifically, granulocytic MDSCs have been demonstrated to suppress T cell-mediated immunopathology in HBV by depriving T cells of L-arginine [124]. MDSC have also been reported to be induced during chronic HCV infection and exert their immunomodulatory role through similar mechanisms [123].

#### 2.2.5. Dendritic Cells in Chronic Viral Hepatitis

Dendritic cells (DCs), which are the main drivers of priming adaptive immune responses to infections, are among the most professional antigen-presenting cells (APC) [125]. They constantly take up foreign antigen and have the potential to present through MHC class I and II, thus priming both, CD4 and CD8 T cell responses. Additionally, through co-stimulatory molecules, including CD80 (B7.1) and CD86 (B7.2) and high-level secretions of IL-12, they activate naïve CD4 and CD8 T cells [126]. DCs comprise at least two main subpopulations with additional subclasses. Myeloid DC, although representing the dominant subpopulation of DCs in the blood, only make up 0.5% of the total PBMC. They constitute of two distinct populations, expressing either CD1c (BDCA-1) or CD141 (BDCA-3) as well as HLA-DR/CD11c/CD1c or CD11c/CD141. mDCs localize to tissue, where they internalize antigen and prime T cell responses [127]. Plasmacytoid DCs comprise an even smaller population of PBMC, averaging about 0.2%. They are expressing CD303 (BDCA-2) or CD123 on lineage negative, CD11c negative HLA-DR-expressing cells. Even though they are able to present antigen, their role is predominantly the production of IFNα in response to the sensing of pathogens by PAMPs [127]. In the liver however, DCs are accompanied by liver sinusoidal endothelial cells (LSEC), which also express CD54, CD80, CD86 and MHC class I and II, thus functioning as APC for CD4 and CD8 T cells as well [128]. Under normal conditions, liver-resident DCs have an immature phenotype, as indicated by low-level expression of MHC class I and II as well as CD86. In liver biopsies of HCV-infected patients, this phenotype is shifted towards a more mature phenotype [129] and in vitro, DCs have been shown to mature in the presence of GM-CSF and HBV antigen [130], thus suggesting a functionally active phenotype during infection. However, data on maturation ability of DCs in the context of HCV infection are still conflicting and further work is required to clearly dissect the role of DCs in chronic HCV infection [131,132,133,134]. In chronic HBV infection, pDCs have been shown to differ significantly from those of healthy donors, in that TLR9-mediated IFNα production is impaired in chronic HBV infection [135]. The mechanism for this is however largely unclear.

#### 2.2.6. Extracellular Vesicles in HBV and HCV Innate Immune Responses

Exosomes are cell-derived vesicles, which have been shown to carry various molecular constituents of their origin cell. Through membrane vesicle transport, exosomes can transfer proteins, lipids and nucleic acids between different cell types. Recently, it has been demonstrated that HBV- and HCV-infected hepatocytes interact with and stimulate DCs via secretion of viral nucleic acid-containing exosomes [136,137]. These exosomes have been demonstrated to transfer IFN-induced antiviral activity to neighboring cells, bypassing the requirement for JAK/STAT signal transduction by directly containing ISG [137,138]. Furthermore, exosomes can transfer infectious HBV and HCV genomes to neighboring cells, resulting in receptor-independent entry [139,140,141,142]. In addition to being secreted from infected hepatocytes, exosomes derived from macrophages during HCV infection were shown to contain microRNA (miRNA)-29 family members, which inhibits HCV replication [143]. Even though it has not yet been shown for HBV or HCV infection, virions themselves can act as transport vesicles for immune activators, including cGAMP, which induces IFN responses in cells following entry [144].

### 2.3. Adaptive Immune Responses

Additionally, cell-based adaptive immune responses against HBV and HCV have been investigated for years. These include CD4 and CD8 T-cells, regulatory T-cells and B-cells (Figure 3).

#### 2.3.1. T Cell Responses to HBV and HCV

The outcome of both, HBV and HCV infection is guided by a combination of genetic factors and virus-specific immune responses. Early studies in chimpanzees and observational studies in humans have identified virus-specific CD4 and CD8 T cell responses as important mechanisms for protection against persistence, even though they are delayed in most cases, probably due to the tolerogenic environment of the liver.

Specifically, the magnitude and breadth of CD4 and CD8 T cell response seems to be a key determinant for spontaneous clearance of HCV infection. During the progression from acute to chronic HCV infection, antigen-specific CD8 T cells are recruited to the liver and nearly disappear from peripheral circulation. Similarly, broadly reactive CD4 T cells are predominantly detectable during acute infection, whereas their numbers decline during chronic infection. Both, CD4 and CD8 T cell responses are deterministic of whether an acute infection is resolved, or whether it progresses to chronic infection. In vivo studies in chimpanzees demonstrated a clear association of CD4 T cell help in maintaining functional CD8 T cell responses, since depletion of CD4 T cells resulted in a decline of antigen-specific CD8 T cell numbers and their functionality.

In chronic infection, the HCV-specific CD4 and CD8 T cells are characterized by high expression of several exhaustion markers, including T cell immunoglobulin and mucin domain (Tim-)3, cytotoxic T lymphocyte-associated antigen-(CTLA)4 and 2B4, which restricts their polyfunctionality, ability to proliferate and degranulate in response to cognate antigen. This includes key effector cytokine production, including interleukin (IL)-2, interferon (IFN)-γ, TNF-α and the degranulation marker CD107a and prevents terminal differentiation to long-lived, CD127-positive memory T cells [145]. Recently, it has been shown in human and chimpanzee studies that this exhausted phenotype is not reverted by treatment with immune checkpoint inhibitors, including antibodies directed against PD-1 [146,147,148,149]. This is a clear result from the prolonged exposure to high levels of viral antigen since CD8 T cells with antigen-specificity for a mutated HCV epitope exhibit lower expression levels of PD-1 and increased levels of responsiveness.

In addition to these classical effector T cell populations, CD4^+^CD25^+^FoxP3^+^ regulatory T cells (T_REG_) have been shown to be enriched in HBV- and HCV-infected patients and actively suppress immune responses to HBV and HCV [150]. This feature of T_REG_ is extending beyond active infection since even T_REG_ from patients following spontaneous clearance are able to suppress HBV-specific CD8 T cell responses. Additionally, HBcAg-specific T_REG_ secreting IL-10 contributes to the suppression of IFNγ in CD4 T cell populations and depletion of T_REG_ restores IFNγ secretion ex vivo [151,152]. This partial restoration of HBcAg-specific T cell responses has also been observed in HBV-infected patients on antiviral treatment, where Adefovir- and Entecavir-treated patients exhibited a decrease in circulating T_REG_ upon viral load suppression [153,154]. In HCV infection, T_REG_ have been shown to be enriched and able to suppress the secretion of IFNγ from HCV-specific CD8 T cells, indicating a role for T_REG_ in the development of chronic infection [155].

Strikingly, this exhausted T cell phenotype is also conserved in tissue-resident T cells (T_RM_), which have recently been shown to be enriched in the liver of patients with chronic HBV infection. These cells, which are characterized by high levels of expression of CD69 and presence or absence of αEβ7 integrin (CD103), consist of a heterogeneous population of memory T cells, including mucosal-associated invariant T (MAIT) cells and γδ T cells. Especially the population of CD69^+^CD103^+^ T_RM_ cells is greatly increased in the liver of HBV-infected patients, accounting for more than 20% of total memory CD8 T cells, compared to 10% in uninfected donors. Even though this population expresses high levels of PD-1, they readily produce IFNγ, IL-2, and TNFα upon restimulation [156].

#### 2.3.2. B Cell Responses in HBV and HCV

Similar to the T cell responses, B cell and humoral responses to infection with HCV are delayed, averagely peaking 6–8 weeks following detection of HCV RNA levels in serum. In contrast to T cell responses, which correlate with spontaneous clearance, there is no association with the development of a robust antibody response and clearance of HCV, since in humans who spontaneously clear infection; antibody responses are often delayed and low in titer. Even though similar to other infectious diseases, including HIV, HCV sometimes elicits the development of broadly neutralizing antibodies (bnAb), these usually are not protective [157]. Even though it has been shown, that these bnAbs are able to control infection in vitro, very limited evidence for the establishment of sterilizing immunity is available [158]. Thus, HCV is almost uniformly able to re-infect patients upon spontaneous or treatment-induced viral clearance.

In contrast, humoral immune responses to HBV infection as well as HBV immunization are predominantly protective and elicit sterilizing immunity. Especially in birth dose vaccinations of HBV, studies have shown 90% of immunized children to exhibit high levels of protective antibodies and that antibody protection against HBV infection is long-lived at over 20 years [159,160]. Only 25% of immunized exhibited antibody levels deemed below protective levels after 22 years of initial vaccination [161]. During natural HBV infection, antibody-mediated protection has been shown to be sterilizing, if production rates of HBsAg-containing sub-viral particles are low, thus facilitating neutralization of Dane particles, or if anti-HBV antibody production is fast and of high affinity [161].

## 3. Immune Evasion Strategies of HCV

### 3.1. Viral Proteins and Their Role in Immune Evasion

With HCV replication restricted to extranuclear compartments, recognition of the uncapped HCV RNA genome or partially double-stranded RNA regions via RIG-I or MDA5 are a constant threat to the virus [57,162,163]. To circumvent the RIG-I/MDA5-initiated induction of interferon regulatory factor (IRF)3 and subsequent production of IFN, the HCV protease NS3/4a has evolved to exert a dual function. In addition to processing and cleaving the HCV polyprotein, NS3/4a rapidly cleaves the adapter protein MAVS, which is essential for relaying RIG-I/MDA5-initiated signal transduction as well as TRIF, which is a downstream signaling mediator of Toll-like receptor (TLR)3, which recognizes double-stranded RNA within endosomal compartments [164,165]. This, in turn, prevents the expression of type I and III IFN [166]. Additionally, NS3 itself was shown to bind tank binding kinase (TBK)1, thus preventing engagement of IRF3 and expression of IFN (Figure 1).

To circumvent the response to paracrine IFN, HCV core has evolved to antagonize and attenuate the JAK/STAT signaling pathway by directly interacting with STAT1. This binding prevents its phosphorylation and thus, activation. Furthermore, HCV core is able to specifically up-regulate SOCS3, thus down-regulating JAK/STAT activity [167]. Moreover, the NS4b protein of HCV efficiently targets STING-mediated TBK1 activation, suppressing the induction of type I IFN [168]. Most notably, HCV has evolved an elaborate structure to bypass host innate immune sensing. Since HCV RNA replication is a prime accumulation of PAMPs for RNA sensors, including RIG-I and MDA5, the complete HCV replicase complex is enveloped in a unique structure termed the membranous web. This structure, which is formed by viral nonstructural proteins from the endoplasmatic reticulum serves as a shield against host responses. It has been recently shown that each of the double-membrane vesicles of the membranous web is sealed by a nuclear pore, which restricts entry of PRR and prevents early signaling, which would result in interferon production [169] (Figure 1).

Other HCV proteins have evolved antagonizing functions against late ISG, including NS5a, which can bind to 2′,5′-OAS, blocking its antiviral effect and reducing ISG expression levels [170]. NS5a has also been implicated in directly binding to the TLR adaptor MyD88 to inhibit TLR-mediated innate immune evasion as well as to protein kinase (PK)R [171]. PKR has been shown can be inhibited by the envelope protein E2 [172] (Figure 1).

### 3.2. Viral Variability and Immune Evasion

The decline in T cell responses, as well as the overall exhausted phenotype of T cells in chronic HCV infection, is thus far poorly understood. Models of HCV kinetics suggest that up to 10^12^ virions are being produced daily [173,174]. HCV NS5b as RNA-dependent RNA polymerase without proofreading capability results in an extensive accumulation of viral quasi-species with a mutation rate of 10^−3^ nucleotides per year [174]. Even though many of these are not functional, HCV has developed a remarkable ability to introduce mutations into various structural and non-structural proteins, which results in replication-competent virus [174]. Mutation of MHC class I and II restricted T cell epitopes may affect the outcome of infection by delaying clearance of HCV-infected cells and may greatly contribute to loss of functionality in T cell responses directed against HCV [175]. Even though this impaired T cell receptor engagement facilitates immune escape, there should be sufficiently novel T cell repertoire to account for these novel antigens. However, it was shown that a substitution in an amino acid of a variant/A*0201 complex results in unaltered binding affinity with significantly impaired ability to expand primed T cells and poor activation of previously primed antigen-specific T cells [176,177].

### 3.3. Evasion of Humoral Immune Responses

The HCV life cycle, in contrast to other viruses, is characterized by two distinct viral egress pathways. In addition to the release of viral particles into the extracellular space, HCV particles can directly cross tight junctions, infecting neighboring cells without the need to diffuse. This, in turn, prevents HCV virions being exposed to antibodies, which may neutralize infectivity. To date, the exact mechanisms of this cell-to-cell spread remain poorly understood, however, cellular lipids have been shown to play an important role during this process [178]. A second mechanism, by which HCV efficiently evades antibody neutralization, is via shifting glycosylation patterns on the E2 glycoprotein [179].

### 3.4. Viral Mimicry of Cellular Lipids

In contrast to other members of the *Flaviviridae*, HCV infectious particles are neither uniform in size nor symmetric in architecture. HCV has evolved assembly and maturation steps in close proximity to cellular lipid droplets. The majority of the infectious HCV particle is comprised of host cellular lipids, including apolipoproteins (Apo)E, A1 and B. Only very few viral antigen and glycoproteins are exposed on this so-called lipoviral particle, potentially to limit exposure of viral antigen to the immune system. Even though ApoE incorporation into HCV particles prevents antibody recognition and enhances infectivity [180], it has been demonstrated that antibodies against ApoE can successfully neutralize HCV infectivity, furthermore underlining the importance of host cell-derived lipids in the HCV life cycle.

## 4. Immune Evasion Strategies of HBV

### 4.1. Viral Proteins and Their Role in Immune Evasion 

Encoding for only eight proteins on six mRNA transcripts, most HBV proteins have evolved ways to impair immune recognition. Initially, to protect the rcDNA from PRR recognition, the HBV capsid does not disintegrate following fusion with the endosome but is actively imported into the nucleus. In contrast to HCV, all coding nucleic acids of HBV are capped and polyadenylated, preventing their recognition by RIG-I or MDA5. However, regions of the HBV pgRNA are double-stranded and may interact with dsRNA sensors. To counteract this, HBV is inducing parkin and the linear ubiquitin assembly complex (LUBAC), which in turn attenuates signal transduction through MAVS [181]. At least two distinct tolerogenic mechanisms can be ascribed to HBeAg and HBsAg. HBeAg, which is dispensable for viral replication, is secreted from infected cells and has been demonstrated to cross the placenta, leading to neonatal tolerance, suppression of antibody and T cell responses to HBcAg, with which it shares sequence homology. In contrast, HBsAg, which comprises the glycoproteins S, M and L of HBV, is secreted from infected cells as infectious Dane particle as well as sub-viral particles. These sub-viral particles are produced in vast excess and are a potent tolerogen. These two secreted viral factors are produced to such high levels that they mask the corresponding antibodies through exhaustive binding. Additionally, both HBeAg and HBsAg have been implicated in impairing TLR signaling initiation. This is orchestrated by HBeAg or the corresponding intracellular p22 form binding directly to MyD88 and TIRAP and interfering with TLR2 signaling. HBsAg has been demonstrated to inhibit TLR2 and c-Jun N-terminal protein kinase (JNK), thus preventing the production of interleukin (IL)-12 [182]. Another important role for HBsAg lies in the subversion of TLR3 signaling since it was demonstrated that HBV elicits type I IFN responses in the absence of HBsAg. Additionally, HBV polymerase actively inhibits both, importin α5 and protein kinase C-δ, thus preventing activated STAT1/2 heterodimers to enter the nucleus to transactivate ISG expression [183]. This points to an active interference with most PRR responsible for recognizing HBV infection. Moreover, this IFN induction may contribute to the immune control of HBV infection upon successful treatment-induced HBsAg seroconversion. Both HBsAg and HBeAg furthermore inhibit the interaction of major vault protein (MVP) with MyD88 in infected cells, thus preventing type I IFN responses [184] as well as inhibiting the action of the ISG tetherin, which usually prevents virion budding [185] (Figure 1).

In addition to these extracellular immune evasion mechanisms, HBV proteins have also been shown to directly interact with host innate immune activation mechanisms. The HBV polymerase, in addition to functioning as reverse transcriptase for pregenomic (pg)RNA, has been shown to directly interfere with PRR signaling via interaction with DDX3 and subsequent inhibition of TBK1 [186]. Additionally, HBp is able to directly interfere with STING-mediated induction of DNA sensing within infected cells [187] and prevent the activation of IKKs through interaction with HSP90β [188]. Furthermore, HBVp masks the antigenic step of reverse transcription by delaying reverse transcription until the HBV pol/pgRNA complex is successfully encapsidated (Figure 1).

A novel immune evasion pathway by HBV has been recently described, which exploits the targeting of interferon regulatory factor (IRF) 3 and 7 nuclear translocation via the NFκB essential modulator (NEMO) by the RUN domain Beclin-1 interacting cysteine-rich-containing (RUBICON) protein, which is driven by HBx [189] (Figure 1).

The HBx protein has been associated to a plethora of biological functions, including competitive inhibition of MAVS-, TRIF- and IRF3-mediated dsRNA recognition through their proteasomal degradation [190]. Additionally, HBx has been implicated in the evasion of transcriptional repression of HBV cccDNA via the structural maintenance of chromosome 5/6 complex (Smc5/6) by targeting it for degradation [191] as well as in inhibiting the transcription of TRIM22, an ISG with anti-HBV capacity [192]. However, many of the HBx-associated mechanisms of innate immune evasion stem from overexpression studies. The HBV proteins core and pre-core can inhibit the expression of MxA in a transcriptional level based on performed in vitro experiments [193] (Figure 1).

### 4.2. Viral Mimicry of HBV cccDNA as Minichromosome

An important step within the HBV life cycle is the conversion of HBV rcDNA to cccDNA, whereby the covalently linked HBV polymerase, as well as an RNA linker, are removed and the incomplete (−)-strand of HBV is repaired [194]. During this process, the HBV genome is organized on host-derived histones [195]. Transcriptional regulation of HBV has been shown to be regulated on an epigenetic level similar to gene regulation on mammalian genes [196]. However, the HBx and HBc proteins play a role in shaping the transcriptional landscape of the HBV genome [197,198]. It has been demonstrated that host factors, including the SETDB1 histone methyltransferase, actively repress HBV transcriptional activity by decreasing histone H3 acetylation and increasing H3K9 tri-methylation [199]. This process is however rapidly reversed by HBx binding to cccDNA and inducing complete transcriptional activation [199]. This host mimicry in combination with HBV transcripts being indistinguishable to host mRNA due to the capped structure and polyadenylation are the main reasons for the extensive stability of HBV cccDNA and absence of innate immune sensing [194]. Even though several cellular mechanisms have been proposed, which could limit the number of cccDNA molecules, most notably the deaminase function of APOBEC3 family members, their role in actually affecting cccDNA quantity in vivo is still highly controversial [200,201,202,203,204,205] (Figure 2).

### 4.3. Evasion of Innate and Adaptive Immune Responses 

In addition to shaping the innate immune activation landscape of HBV-infected hepatocytes, HBV has furthermore been shown to affect a number of other, liver-resident cell types. HBeAg, which is secreted by HBV-infected hepatocytes, is impairing TLR2 expression levels in KCs, thus suppressing their ability to respond to cell-wall components, lipoteichoic acid, and lipoproteins [121,206]. Furthermore, macrophages exposed to HBsAg and HBcAg selectively inhibit TLR2 activation, limiting IL-12 secretion and contribute to CD8 T cell exhaustion via a direct interaction between HBcAg and TLR2, respectively [120,182]. An additional interaction between HBcAg and gC1qR further suppresses TLR4-induced IL-12 production [207]. These mechanisms have been shown to be particularly important for the induction of tolerance in vertical transmissions of HBV, where maternally derived HBeAg enhances PD-L1 expression in macrophages of offspring to suppress the induction of CD8 T cell responses [208].

NK cells, which can take up HBV nucleic acids through exosomes, exhibit an impaired phenotype in HBV infection, since IFNγ production, cytotoxic activity, proliferation and responsiveness to exogenous stimuli is reduced, compared to healthy donors [139]. Even though it has been shown that exosomes from HBV-infected hepatocytes contain rcDNA, sub-genomic HBV RNA and HBV proteins, the exact mechanism for this are still unclear. Another feature of the interaction of HBV with NK cells is their general frequency, activation and cytokine production, which is significantly reduced in HBV-infected patients compared to healthy controls with especially the anti-inflammatory cytokine IL-10 being elevated [209,210]. Additionally, NK cells of HBV-infected patients are characterized by elevated expression levels of inhibitory receptors and reduced expression of activating receptors [211,212,213]. This could explain the altered responses of NK cells in HBV infection.

Another method of evading immune detection of HBV is the selective loss of HBeAg through the accumulation of mutations within the pre-core or basal core promoter [214]. This loss of HBeAg, which usually occurs during the inactive carrier phase of infection, removes one of the key targets for the immune response, effectively concealing HBV from immune recognition [12].

## 5. Conclusions

HBV and HCV as hepatotropic viruses have adapted efficiently to survive within the liver, where the highly tolerogenic environment further facilitates viral persistence. This sets HBV and HCV apart from other infectious diseases, which persist in less immune-privileged sites within the body. Despite the overall similar disease progression, where liver cirrhosis and hepatocellular carcinoma often define the endpoint of infection, HBV and HCV have evolved distinct means of evading or subverting the host response. Overall, HBV is more immune silent compared to HCV, simply by the reduced number of PAMP present in the viral genome. Most of the HBV life cycle remains hidden from the host by physically locating it within assembled viral capsids. Additionally, HBV has evolved all its transcripts to resemble cellular mRNAs, further reducing the likelihood of immune recognition. HCV, on the other hand, encodes a multitude of PAMPs, ranging from its uncapped 5′ internal ribosomal entry site (IRES) structure, partially double-stranded RNA genome segments and lack of polyadenylation signal. These features would suggest, that HBV actively interferes with immune activation, rather than remaining hidden from immune detection. However, this is still controversial and evidence for both hypotheses have been presented. However, since HBsAg, which is also used as HBV vaccine, is highly immunogenic [215,216,217], the nearly complete absence of immune responses during early HBV infection still remains elusive. Further study of the interaction of HBV and the immune system in physiological model systems are therefore required.

To counteract the inevitable activation of cytoplasmic RNA sensors, which have specialized in recognizing these PAMP, HCV has adopted more direct means of immune evasion. This includes a crippling of the RNA-mediated IFN responses by disrupting signaling through TLR3, RIG-I, and MDA5 via its protease. Replication of HCV RNA, which is another foreign process in cells, is hidden from the host cell by enveloping itself in the membranous web, further underlining the plethora of changes, which HCV induces in infected cells to hide from immune recognition. In contrast, steps during the HBV life cycle, which carry a significant risk of detection by the immune system, are well hidden within the viral capsid. However, this concealment is virally encoded and does not require extensive restructuring of infected cells. Nevertheless, both viruses encode proteins, which the host may recognize as being foreign. Over the past years, it has been more and more acknowledged that HBV, in addition to HCV, modulates early events during infection in order to restrict this from happening. This has gradually shifted the view on HBV from a pathogen, which interacts very limited with the innate immune system, to one, which expertly subverts recognition. Additionally, the HBV has been shown to interact with a wide range of other immune cells to maintain the initial immunotolerant state after infection.

Even though several interferon effector molecules have been described to affect infection of HBV or HCV, many of the studies have been performed using overexpression of ISG. However, endogenous IFN-mediated induction of many ISG is resulting in much more subtle expression changes. Thus, it remains an open question as to how much these ISG contribute to viral clearance over simply delaying virus replication until adaptive immune responses can be mounted.

Once chronic infection is established, both, HBV and HCV interfere with adaptive T cell responses, resulting in the gradual emergence of exhausted T cell phenotypes. Even though current interventional studies are aimed at breaking T cell tolerance, the exact mechanisms, by which T cell exhaustion occurs and is maintained, require further investigation. In an era of direct acting antiviral drugs able to eradicate HCV from the liver, studies are needed to evaluate the long-term impact of HCV antigen removal on T cell exhaustion. This can potentially result in important insights, which may be translatable to HBV infection.

To conclude, multiple pathways, by which HBV and HCV evade innate and adaptive immune recognition have been described. Nevertheless, important open questions in regards to why HBV infection does not induce early innate immune activation, the detailed mechanisms of action of ISG against HCV or HBV, whether HBV cccDNA can be exposed to immune recognition, the inability of HCV to induce sterilizing immunity and the mechanism of HBV- and HCV-associated T cell exhaustion still requires further investigation.

## Figures and Tables

**Table 1:** Interferon-stimulated genes with activity against HBV and HCV.

Interferon-Stimulated Gene	ISG Class	Effect on HCV	Effect on HBV	Reference
ADAP2	Direct antiviral	Inhibits entry	-	[20]
ADAR	Direct antiviral	RNA editing	-	[21]
APOBEC1, 3A, 3B, 3G, 3F	Direct antiviral	-	cccDNA/HBV DNA editing	[22,23,24,25,26,27]
AID	Direct antiviral	-	cccDNA editing	[28,29,30]
CH25H	Unknown	Inhibits replication	Inhibits entry	[31,32,33,34]
DDIT4	Unknown	Inhibits infection	-	[21]
DDX58 (RIG-I)	RNA sensor	Enhanced viral sensing	Inhibits replication	[21,35,36,37]
DDX60	RIG-I enhancer	Inhibits infection	-	[21,38,39]
DDX60L	Unknown	Inhibits replication	-	[40]
EIF2AK2 (PKR)	RNA sensor	Inhibition of translation	Inhibition of replication	[41,42,43,44]
GBP1	Unknown	Inhibits infection	-	[45]
IFI44L	Unknown	Inhibits infection	-	[21]
IFI6	Unknown	Inhibits HCV entry	-	[45,46]
IFI27	Unknown	Inhibits infection	-	[21]
IFIT1	Direct antiviral	Sequesters viral nucleic acids	-	[45,47]
IFIT3	MAVS/TBK1 enhancer	Inhibits infection	-	[48]
IFITM1	Direct antiviral	Inhibits entry and replication	Inhibition of replication	[48,49,50,51]
IFITM3	Direct antiviral	Inhibits translation	-	[52]
IRF1	ISG inducer	Inhibits infection	Inhibition of replication	[21,44,53]
IRF2	Unknown	Inhibits infection	-	[21]
IRF7	IFN inducer	Inhibits infection	-	[21]
IRF9	IFN inducer	Inhibits infection	-	[54]
ISG12	Ubiquitin-dependent degradation	Viral protein degradation	-	[45]
ISG20	Direct antiviral	Cleaves RNA genome	Inhibits encapsidation, Cleaves RNA genome	[42,55,56]
ISG56	Unknown	Inhibits replication	-	[49]
MAP3K14	Unknown	Inhibits infection	-	[21]
MDA5	RNA sensor	Inhibits infection	-	[21,57]
MOV10	Unknown	Inhibits infection	-	[21]
MS4A4A	Unknown	Inhibits infection	-	[21]
MX1	ISG inducer	Inhibits infection	Inhibits replication	[45,58,59]
MYD88	IFN inducer	-	RNA degradation	[60]
NOS2	Unknown	-	Inhibits replication	[48,61]
NT5C3	Unknown	Inhibits infection	-	[21]
OAS1	RNase I activator	Inhibits infection	-	[45,62]
OASL	Unknown	Inhibits replication	-	[21,63]
PLSCR1	Unknown	Inhibits infection	Inhibits replication	[48,64]
RNASEL	Direct antiviral	Cleaves RNA genome	Inhibition of replication	[44,48]
R2AD2	Direct antiviral	Inhibits replication	-	[48]
STING	IFN inducer	-	Inhibits HBV assembly	[65]
SOCS3	IFN negative regulator	Inhibits replication	-	[66,67]
STAT1/3	IFN inducer	Inhibits replication	Inhibits transcription	[68,69,70]
BST2 (Tetherin)	Direct antiviral	Inhibits HCV secretion	Inhibits HBV secretion	[71,72,73]
TLR3	RNA sensor	Inhibits infection	Inhibits replication	[74,75,76]
TLR7	RNA sensor	Inhibits infection	Inhibits replication	[77,78,79]
TLR8	RNA sensor	Inhibits infection	Inhibits replication	[78,79]
TRIM14	Unknown	Degradation of NS5a	-	[48,80]
TRIM12a	Unknown	Inhibits infection	-	[21]
TRIM22	Unknown	Ubiquitination of NS5a	Inhibits transcription	[81,82]
TRIM41	E3 ubiquitin ligase activator	-	Inhibits transcription	[83]
Viperin	Direct antiviral	Inhibits replication	-	[84]
ZAP	Unknown	-	Inhibits replication	[85,86]
